# Case Report: Dynamic TKI combination strategies for EGFR-mutant NSCLC with acquired ROS1 fusion and brain metastases

**DOI:** 10.3389/fonc.2026.1825642

**Published:** 2026-06-04

**Authors:** Hua Yang, Yong Da, Ge Shen, Qi-Yun Sun, Zhi-Yu Yao, Ya-Jing Zhou

**Affiliations:** Department of Oncology, Beijing Fengtai You’anmen Hospital, Beijing, China

**Keywords:** case report, EGFR, non-small cell lung cancer, resistance, ROS1

## Abstract

The development of a ROS1 fusion as a bypass signaling pathway mediating resistance to third-generation EGFR tyrosine kinase inhibitors (TKIs) is rare, with only sporadic cases reported in the literature. Consequently, no consensus exists on the optimal treatment sequencing for such patients. We report the case of a 61-year-old woman diagnosed with stage IV lung adenocarcinoma with brain metastasis harboring an EGFR exon 19 deletion (p.E746_A750del), who acquired resistance to osimertinib through a ROS1 fusion bypass pathway. The patient achieved a survival of over 6.5 years from initial diagnosis through ongoing adjustments to targeted therapy, including sequential treatment with crizotinib, entrectinib, and lorlatinib. Although all three agents target ROS1, they exhibited distinct side effect profiles in this patient. Notably, we observed an interesting phenomenon with both crizotinib and entrectinib: while initial treatment led to intolerable adverse reactions requiring discontinuation, subsequent reintroduction of the same agent was well-tolerated. This case report aims to provide potential treatment strategies for patients with similar complex co-mutations.

## Introduction

Approximately 50% of patients with non-squamous non-small cell lung cancer (NSCLC) present with actionable oncogenic alterations. The prevalence of ROS1 gene fusions in Asian and Asia-Pacific NSCLC populations is approximately 3% (1). The emergence of ROS1 bypass pathway as a resistance mechanism to third-generation EGFR TKIs is exceedingly rare. The co-occurrence of a sensitizing EGFR mutation and a ROS1 fusion presents a significant therapeutic challenge, underscoring the need for individualized treatment approaches. Herein, we report the case of an NSCLC patient with an EGFR exon 19 deletion (p.E746_A750del) and brain metastases who acquired resistance to osimertinib via a ROS1 fusion bypass pathway. Through dynamic adjustments to targeted therapy, the patient has achieved a survival of 78 months from initial diagnosis.

## Case presentation

### Diagnostic assessment

In August 2019, a 61-year-old woman with no smoking history or family history of cancer presented to our hospital with a one-month history of cough and chest pain. Computed tomography (CT) and positron emission tomography-CT (PET/CT) revealed a mass lesion in the dorsal segment of the left lower lobe, measuring approximately 2.5 × 2.8 cm, accompanied by multiple small pulmonary nodules, multiple liver metastases, and multiple bone metastases, including a large lesion in the right iliac bone and a C5 vertebral lesion with suspected spinal canal invasion. Additional findings included a right frontal lobe mass, a right retroperitoneal fat space metastasis, multiple lymph node metastases in the mediastinum, left hilum, and neck, and invasion of the superior vena cava with possible thrombosis. The clinical stage was cT1c, cN3, cM1c (stage IVB) (**Figure 1a**). Initial molecular profiling (August 2019) was performed using two complementary methods. EGFR exons 18, 19, 20, and 21 were tested by reverse transcription polymerase chain reaction (RT-PCR), which revealed an EGFR exon 19 deletion (c.2235_2249del, p.E746_A750del). Concurrently, a next-generation sequencing (NGS) assay using a hybrid capture-based panel was performed to assess KRAS exons 2–4, BRAF exons 11 and 15, and the ALK fusion locus, all of which were negative. The NGS assay had a sequencing depth of 1445 base pairs, and the tumor proportion in the biopsy specimen was approximately 40%. At the time of initial diagnosis, this NGS panel did not routinely assess other co-alterations such as TP53, PIK3CA, CDK4/6, CDKN2A/B, copy number variations (e.g., MET, ERBB2, EGFR amplification), or tumor mutational burden (TMB). The patient’s treatment course is delineated into three phases: single-target therapy, chemotherapy, and dual-target therapy (**Figure 2**).

**Figure 1:**
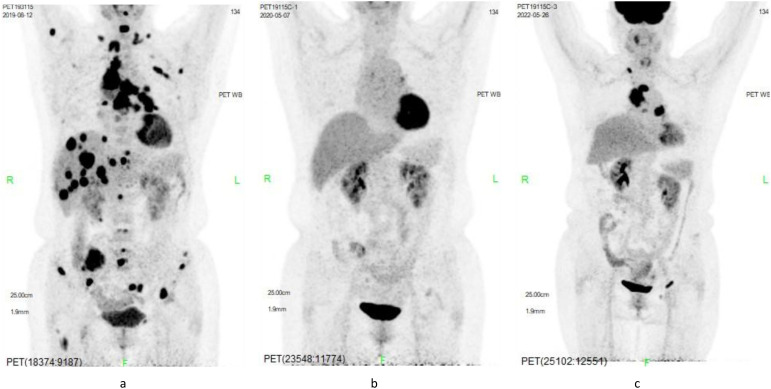
Radiographic findings at diagnosis and upon progression on first-line TKI. PET-CT images at key time points. **(a)** At initial diagnosis (August 2019), showing the primary left lower lobe lesion, multiple metastases, and a right frontal lobe brain metastasis. **(b)** During treatment with icotinib, demonstrating disease control. **(c)** At the time of resistance to icotinib (May 2022), revealing disease progression with new lesions in the mediastinal lymph nodes, right supraclavicular lymph node, right parietal lobe, and right cerebellum.

**Figure 2:**
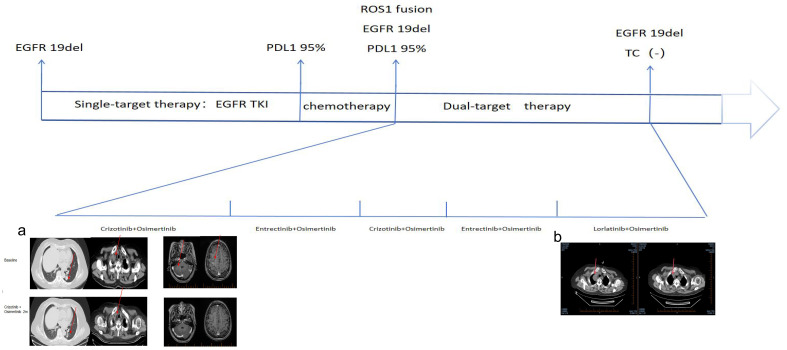
Treatment timeline, radiologic responses, and molecular guidance. Treatment and molecular testing timeline. A chronological overview of the patient’s 78-month treatment journey, divided into single-target therapy (osimertinib, icotinib), chemotherapy (bevacizumab/pemetrexed/carboplatin), and dual-target therapy phases (crizotinib+osimertinib, entrectinib+osimertinib, lorlatinib+osimertinib). Key molecular testing time points and results (EGFR 19del, ROS1 fusion, EZR-ROS1 fusion, PD-L1 expression) are annotated to illustrate their role in guiding sequential therapeutic decisions. **(a)** Radiologic response to first dual-target therapy (crizotinib + osimertinib). Computed tomography (CT) scans comparing disease burden before (left) and two months after (right) initiating crizotinib plus osimertinib. Images demonstrate a significant, marked shrinkage of extracranial lesions, showing a profound initial response to the dual-targeted regimen. **(b)** Radiologic response to third-line dual-target therapy (lorlatinib + osimertinib). CT images of the new right lung apex lesion that developed after progression on entrectinib. Left panel: Baseline image before starting lorlatinib plus osimertinib. Right panel: Follow-up image after 20 days of treatment, demonstrating a clear partial response of the lesion.

### Single-target therapy phase (August 2019-May 2022)

Given the excellent central nervous system (CNS) penetration of third-generation EGFR TKIs, the patient was initially started on osimertinib 80 mg once daily (qd) orally. After two months, imaging demonstrated complete resolution of intracranial lesions and significant shrinkage of extracranial lesions. Due to cost considerations, the patient was transitioned to icotinib 125 mg three times daily (tid) from November 2019 to May 2022. Treatment was discontinued in May 2022 due to disease progression in the mediastinal and right supraclavicular lymph nodes, as well as new lesions in the right parietal lobe and right cerebellum (**Figure 1c**). The patient underwent radiotherapy to the mediastinal lymph nodes from May 25th to June 22nd, 2022 (46 Gy in 20 fractions, 230 cGy/fraction). On July 13th, 2022, an excisional biopsy of the right supraclavicular lymph node was performed. Postoperative pathology confirmed lymph node metastasis of lung adenocarcinoma, and subsequent NGS testing revealed no driver gene mutations and a programmed cell death ligand 1 (PD-L1) tumor proportion score (TPS) of 95%.

### Chemotherapy phase (July 2022-July 2023)

From July 2022, the patient received eight cycles of treatment with bevacizumab combined with pemetrexed and carboplatin. Treatment efficacy was assessed as stable disease in both intracranial and extracranial lesions. This was followed by eight cycles of pemetrexed maintenance monotherapy. Chemotherapy was discontinued due to disease progression in the right lower lobe lung lesion.

In June 2023, a biopsy of a right lower lobe lung lesion was performed. Targeted reverse transcription-polymerase chain reaction (RT-PCR) revealed an EGFR exon 19 deletion (p.E746_A750del) and a ROS1 rearrangement. Pathology confirmed lung adenocarcinoma, with immunohistochemistry (IHC) results as follows: NapsinA (+), CK5/6 (-), P40 (-), TTF-1 (+), ALK-D5F3 (-), L858R (-), CK (+), synaptophysin (-).

### Dual-target therapy phase (July 2023-January 2026)

Combination therapy with crizotinib (200 mg twice daily) and osimertinib (80 mg daily) was initiated on July 8th, 2023. After two months, imaging showed complete resolution of intracranial lesions and significant shrinkage of extracranial lesions (**Figure 2a**). However, the patient soon developed intolerable adverse effects, including bloating, abdominal pain, anorexia, and bone marrow suppression. Consequently, on November 1st, 2023, the crizotinib dose was reduced to 250 mg once daily. Shortly after this dose reduction, new lesions soon appeared in the right frontal lobe and left occipital lobe. In December 2023, the patient underwent stereotactic radiotherapy to the right frontal lobe lesion (54 Gy in 18 fractions). In March 2024, treatment was switched to entrectinib combined with osimertinib (80 mg daily). A similar pattern to that seen with crizotinib emerged: intracranial lesions completely resolved, but intolerable adverse reactions developed at the standard entrectinib dose of 600 mg daily. After dose reduction to 400 mg daily, the left occipital lobe lesion progressed. In May 2024, the patient received stereotactic radiotherapy to the left occipital lobe lesion (54 Gy in 18 fractions).

In September 2024, crizotinib 200 mg twice daily was restarted. This time, the regimen was well-tolerated. While no new intracranial lesions developed, the left lower lobe lung lesion showed slow progression. On January 1st, 2025, a trial of entrectinib was repeated. Consistent with the prior experience, the adverse reactions were tolerable, and no new intracranial lesions appeared, but the lung lesions continued to progress slowly. On February 12th, 2025, the left lower lobe lesion was treated with CyberKnife stereotactic body radiotherapy (42.5 Gy in 5 fractions).

In May 2025, follow-up CT revealed a new lesion in the apex of the right lung. Biopsy of this lesion confirmed non-small cell lung cancer. NGS testing identified an EGFR exon 19 deletion (p.E746_A750del) with allelic fractions of 34% (sample 1) and 59% (sample 2), and an EZR-ROS1 fusion (exon 10-exon 34) with allelic fractions of 17.5% (sample 1) and 24.8% (sample 2). PD-L1 expression was high (TPS 80%). On July 23rd, 2025, the patient commenced treatment with lorlatinib (100 mg daily) combined with osimertinib (80 mg daily). After seven months of this regimen, the new lesion in the right lung apex demonstrated a partial response (**Figure 2b**).

During subsequent treatment, the patient experienced intolerable grade 2–3 adverse events, prompting a dose reduction of lorlatinib from 100 mg qd to 50 mg qd. Shortly after the dose reduction, the patient developed hemoptysis. Bronchoscopy revealed a new lesion in the left bronchus, and biopsy again confirmed lung adenocarcinoma. Notably, next-generation sequencing (NGS) of this new lesion did not detect the ROS1 fusion but revealed a PTEN p.C124S mutation (allelic frequency 51.11%). Starting January 7th, 2026, the patient began chemotherapy with nab-paclitaxel and cisplatin.

## Discussion

We report a case of an NSCLC patient with an EGFR exon 19 deletion (p.E746_A750del) and brain metastases who acquired resistance to osimertinib via a ROS1 fusion bypass pathway. This case details the 78-month survival of a patient from initial diagnosis. Approximately 50% of patients with non-squamous NSCLC harbor actionable oncogenic alterations, with targeted therapies available for drivers in EGFR, KRAS, HER2, BRAF, MET, ALK, ROS1, RET, and NTRK1–3 genes (2, 3). Classic mutations (exon 19 deletions and exon 21 L858R mutations) typically exhibit more favorable responses to EGFR TKIs than atypical mutations (4), with differences in molecular mechanisms, clinical presentations, treatment outcomes, and prognosis.

Multiple studies suggest that patients with EGFR exon 19 deletions (19Del) achieve higher objective response rates (ORR), disease control rates (DCR), progression-free survival (PFS), and overall survival (OS) with EGFR-TKI treatment compared to those with the L858R mutation, although not all differences reach statistical significance (5). Even in the rare context of concomitant driver alterations, the EGFR 19Del allele can retain sensitivity to EGFR TKIs (6). Given the excellent blood-brain barrier (BBB) penetration of third-generation EGFR-TKIs, osimertinib was selected as first-line targeted therapy. The subsequent switch to icotinib yielded a PFS of 31 months. Recent research has expanded beyond sensitivity to targeted therapy, exploring differences in imaging characteristics, co-mutation patterns, tumor growth dynamics, and metastatic mechanisms between 19Del and L858R mutations (7).

Drug resistance is an inevitable challenge in EGFR-mutant NSCLC. The mechanisms of acquired resistance differ by EGFR-TKI generation. In patients treated with first- or second-generation EGFR-TKIs, the EGFR T790M mutation is the most common resistance mechanism, occurring in approximately 50–60% of cases. In contrast, resistance to third-generation EGFR-TKIs such as osimertinib involves a broader spectrum of alterations, including on-target mutations (e.g., C797S), bypass pathway activation (e.g., MET amplification, HER2 amplification, BRAF mutations, and gene fusions such as ROS1), as well as histological transformation (8–12). In the present case, resistance to osimertinib developed through the activation of a ROS1 fusion bypass pathway rather than through the emergence of a T790M mutation. Following progression on EGFR TKI, the patient experienced a prolonged response (>1 year) to pemetrexed-based chemotherapy. A later biopsy at the time of subsequent disease progression detected a ROS1 rearrangement by RT-PCR. ROS1 (c-ros oncogene 1), a receptor tyrosine kinase involved in regulating cell growth, differentiation, and survival (13), shares high structural homology with ALK (14). Gene rearrangements, fusions, or point mutations can generate constitutively activated oncogenic drivers in various tumors (15). Common ROS1 fusion partners include CD74, SDC4, and SLC34A2, which stabilize the ROS1 kinase domain and promote continuous signaling (16). The incidence of ROS1 rearrangement in NSCLC is approximately 1-2% (17, 18), with a higher proportion in never-smoking Asian patients with lung adenocarcinoma (19). Detection methods include IHC, fluorescence *in-situ* hybridization (FISH), PCR, and DNA/RNA-based NGS, with NGS improving the detection rate of functional fusions (20, 21). The patient’s EZR-ROS1 fusion was identified via NGS.

The coexistence of an EGFR driver mutation with another driver alteration like ROS1 rearrangement is globally rare, and the lack of data precludes a clear consensus on management. Decision-making is influenced by factors such as mutation prevalence and the variant allele frequency (VAF) reported by NGS, with a higher VAF potentially guiding the choice of targeted therapy. Given the comparable VAFs of the EGFR exon 19 deletion (p.E746_A750del) and EZR-ROS1 fusion in this case, a dual-targeted therapy approach combining a third-generation EGFR-TKI and a ROS1-TKI was adopted.

Despite crizotinib’s known limited blood-brain barrier (BBB) penetration (22), its combination with osimertinib led to a complete intracranial response. We hypothesize that osimertinib, with its excellent CNS penetration, may have altered crizotinib’s intracerebral distribution, potentially through drug-drug interactions affecting membrane transporters (23).

Lorlatinib is a third-generation macrocyclic TKI designed to inhibit ALK and ROS1-driven tumors while overcoming resistance mutations and improving CNS penetration compared to earlier TKIs (24). Although FDA-approved for ALK-positive NSCLC, its precise role in ROS1-positive disease is still being defined (25, 26). Real-world analyses from expanded access programs have reported an ORR of 41% and a median PFS of approximately 11.9 months in ROS1-positive patients (27). In the GLASS study, among 17 ROS1-positive patients, extracranial and intracranial response rates were 62% and 67%, respectively, with DCR exceeding 78% for both (28). Although lorlatinib showed promising efficacy, our case also revealed an intriguing tolerability phenomenon shared by crizotinib and entrectinib. Common adverse events include hypercholesterolemia, hypertriglyceridemia, edema, neurocognitive effects, and peripheral neuropathy (29, 30). Real-world studies indicate that approximately 40% of patients require dose reductions due to treatment-related adverse events (TRAEs), primarily neurocognitive or neuropathic, though dose reduction does not appear to impact PFS or OS (31). This latter finding contrasts with our case, where lorlatinib dose reduction was followed by rapid disease progression manifesting as hemoptysis and a new bronchial lesion.

Furthermore, we observed an interesting clinical phenomenon with both crizotinib and entrectinib: although initial treatment was discontinued due to adverse reactions, reintroduction of the same agent at a later time point was well-tolerated. The exact mechanism underlying this observation requires further investigation and more data. Despite all three drugs targeting ROS1, our case demonstrates that switching to an alternative agent can yield significant efficacy when one proves ineffective or intolerable. Importantly, we observed distinct side effect profiles among lorlatinib, crizotinib, and entrectinib in this patient (**Table 1**). For instance, lorlatinib’s primary adverse effects were edema and hyperlipidemia, whereas crizotinib mainly caused bloating, abdominal pain, and anorexia.

**Table 1:** Comparison of adverse event profiles across three ROS1-TKIS.

Therapeutic regimen	AE	Grade
Crizotinib (200 mg bid) + Osimertinib (80mg qd)	Abdominal pain	3
	Bloating	2
	Anemia	2
	Leukopenia	2
	thrombocytopenia	1
Entrectinib (600mg qd) + Osimertinib (80mg qd)	thrombocytopenia	3
	Generalized edema	2
	Anemia	2
	Leukopenia	2
	Blurred vision	1
	Alanine aminotransferase increased	1
Lorlatinib (100mg qd) + Osimertinib (80mg qd)	Ventricular tachycardia	3
	Anemia	3
	thrombocytopenia	2
	Hyperlipidemia	2

A summary of the different adverse reactions experienced by the patient during treatment with each ROS1-TKI (crizotinib, entrectinib, and lorlatinib) when combined with osimertinib. The table highlights the distinct tolerability patterns observed with each agent, such as gastrointestinal effects with crizotinib, and edema and hyperlipidemia with lorlatinib.

We also confirm that the May 2025 NGS assay systematically assessed the entire ROS1 kinase domain, including known resistance mutations such as G2032R, L2026M, F2004C, D2033N, and L2086F. Notably, no ROS1 resistance mutations were detected in either tissue sample. This absence after three lines of ROS1-TKI therapy (crizotinib, entrectinib, and lorlatinib) is indeed notable and suggests that the resistance mechanism in this patient was likely ROS1-independent. Supporting this conclusion, a subsequent biopsy of a bronchial lesion in December 2025 revealed complete loss of the ROS1 fusion and the emergence of a PTEN p.C124S mutation (allelic frequency 51.11%), a known activator of the PI3K/AKT bypass pathway that can confer resistance to both EGFR- and ROS1-TKIs. These findings underscore the importance of repeat biopsy and comprehensive molecular profiling at each progression event.

Nevertheless, several limitations of this case should be acknowledged. First, regarding the negative driver mutation result from the right supraclavicular lymph node biopsy (July 13, 2022): although the biopsy was performed at our institution, the genetic testing was independently arranged by the patient through an external commercial laboratory; therefore, we lack access to the detailed methodology or sample quality control data. Based on subsequent NGS assays (May 2025 and December 2025), which consistently detected the EGFR exon 19 deletion (p.E746_A750del) with allelic frequencies ranging from 26% to 59% across different tumor sites, we conclude that the 2022 negative result most likely reflects technical limitations of the external assay (e.g., low sensitivity or insufficient tumor content) rather than true biological loss of the driver mutation. However, concurrent tumor heterogeneity and clonal evolution under selective pressure from prior EGFR-TKI therapy cannot be completely excluded.

Second, it is also important to acknowledge that liquid biopsy (circulating tumor DNA analysis) was performed at two time points of disease progression (May 2023 and July 2025). However, neither test detected the driver EGFR mutation, ROS1 fusion, or any known resistance mutations—only low-frequency TP53 variants were identified. These negative results highlight a known limitation of ctDNA analysis in certain clinical scenarios: tumors with low shedding of DNA into the bloodstream, particularly those with isolated intracranial or slowly progressing extracranial disease, may yield false-negative results. Consequently, in this patient, repeat tissue biopsy proved essential for deciphering the dynamic resistance mechanisms. This experience underscores that while liquid biopsy is a valuable tool, a negative result should not preclude tissue re-biopsy when clinically indicated.

Compared to previously reported cases, this patient presented with a more advanced disease stage and a higher metastatic burden at diagnosis. Notably, she has survived for 78 months since her initial diagnosis. At critical treatment junctures, timely and comprehensive molecular testing was performed, both to elucidate resistance mechanisms and to offer new therapeutic opportunities. Future research will increasingly focus on optimizing treatment regimens for this unique patient population, aiming to assess the efficacy and safety of different therapies and identify the optimal sequence and combinations.

## Patient perspective

The patient provided her perspective on the 6.5-year treatment journey: “When I was first diagnosed with advanced lung cancer and told it had spread to my brain, I was devastated. But my doctors explained each treatment option clearly and always involved me in decision-making. The targeted therapies allowed me to maintain a good quality of life—I was able to continue caring for my family and even traveled during treatment breaks. The side effects were challenging at times, especially the digestive issues with crizotinib, but I learned to manage them with dietary adjustments and support from my medical team. I am grateful for the time these treatments have given me and hope my story can help other patients facing similar challenges.”

## Data Availability

The original contributions presented in the study are included in the article/**Supplementary Material**. Further inquiries can be directed to the corresponding author.

## References

[1] Meyer ML Fitzgerald BG Paz-Ares L Cappuzzo F Janne PA Peters S . New promises and challenges in the treatment of advanced non-small-cell lung cancer. Lancet. (2024) 404:803–22. doi: 10.1016/s0140-6736(24)01029-8. PMID: 39121882

[2] Hendriks LE Kerr K Menis J Mok TS Nestle U Passaro A . Oncogene-addicted metastatic non-small-cell lung cancer: ESMO Clinical Practice Guideline for diagnosis, treatment and follow-up. Ann Oncol. (2023) 34:339–57. doi: 10.1016/j.annonc.2022.12.009. PMID: 36872130

[3] National Comprehensive Cancer Network . NCCN Clinical Practice Guidelines in Oncology (NCCN Guidelines): non-small cell lung cancer, version 2.2024. Plymouth Meeting (PA: National Comprehensive Cancer Network (2024). Available online at: https://www.nccn.org/guidelines/guidelines-detail?category=1&id=1450 (Accessed May 15, 2026).

[4] Ferro A Marinato GM Mulargiu C Marino M Pasello G Guarneri V . The study of primary and acquired resistance to first-line osimertinib to improve the outcome of EGFR-mutated advanced non-small cell lung cancer patients: the challenge is open for new therapeutic strategies. Crit Rev Oncol Hematol. (2024) 196:104295. doi: 10.1016/j.critrevonc.2024.104295. PMID: 38382773

[5] Zhou J Ben S . Comparison of therapeutic effects of EGFR-tyrosine kinase inhibitors on 19Del and L858R mutations in advanced lung adenocarcinoma and effect on cellular immune function. Thorac Cancer. (2018) 9:228–33. doi: 10.1111/1759-7714.12568. PMID: 29222872 PMC5792717

[6] Liang H Li C Zhao Y Zhao S Huang J Wang W . Concomitant mutations in EGFR 19Del/L858R mutation and their association with response to EGFR-TKIs in NSCLC patients. Cancer Manag Res. (2020) 12:8653–62. doi: 10.2147/cmar.s255967. PMID: 32982456 PMC7509478

[7] Han P Wang Z Li K Liu Y Zhang L Chen H . Noninvasive prediction of EGFR 19Del and 21L858R subtypes in lung adenocarcinoma: a comparative study of logistic regression and decision tree models. Front Oncol. (2025) 15:1642253. doi: 10.3389/fonc.2025.1642253. PMID: 41049833 PMC12488440

[8] Wagener-Ryczek S Heydt C Süptitz J Michels S Falk M Ihle MA . Mutational spectrum of acquired resistance to reversible versus irreversible EGFR tyrosine kinase inhibitors. BMC Cancer. (2020) 20:408. doi: 10.1093/annonc/mdz063.032 32397977 PMC7216404

[9] Wu SG Liu YN Tsai MF Chang YL Yu CJ Yang PC . The mechanism of acquired resistance to irreversible EGFR tyrosine kinase inhibitor-afatinib in lung adenocarcinoma patients. Oncotarget. (2016) 7:12404–13. doi: 10.18632/oncotarget.7189. PMID: 26862733 PMC4914294

[10] Ma C Wei S Song Y . T790M and acquired resistance of EGFR TKI: a literature review of clinical reports. J Thorac Dis. (2011) 3:10–8. doi: 10.3978/j.issn.2072-1439.2010.12.02 PMC3256494 22263058

[11] Ahsan A . Mechanisms of resistance to EGFR tyrosine kinase inhibitors and therapeutic approaches: an update. Adv Exp Med Biol. (2016) 893:137–53. doi: 10.1007/978-3-319-24223-1_7. PMID: 26667342

[12] Gao J Li HR Jin C Jiang JH Ding JY . Strategies to overcome acquired resistance to EGFR TKI in the treatment of non-small cell lung cancer. Clin Transl Oncol. (2019) 21:1287–301. doi: 10.1007/s12094-019-02075-1. PMID: 30864018

[13] Zhang HL Kong Q . Current landscape and future prospects of RET and ROS1 targets. Pharm Pat Anal. (2023) 12:113–26. doi: 10.4155/ppa-2023-0011. PMID: 37671904

[14] Li Y Wang Z Zhang H Liu Y Chen X Zhao J . Recent advances in the development of dual ALK/ROS1 inhibitors for non-small cell lung cancer therapy. Eur J Med Chem. (2023) 257:115477. doi: 10.1016/j.ejmech.2023.115477. PMID: 37210839

[15] Yang X Li D Jiang X Sun Y Zhang L Wang J . Progress of non-small-cell lung cancer with ROS1 rearrangement. Front Mol Biosci. (2023) 10:1238093. doi: 10.3389/fmolb.2023.1238093. PMID: 38187090 PMC10766828

[16] Jeong JS Lee S Park S Kim H Choi YL Ahn MJ . Molecular and clinical characteristics of ROS1 fusion variants in ROS1-rearranged cancers: a defined role of fusion partners and breakpoints of ROS1. Eur J Cancer. (2025) 231:116091. doi: 10.1016/j.ejca.2025.116091. PMID: 41205256

[17] Desilets A Repetto M Yang SR Sherman EJ Jänne PA Drilon A . Targeting ROS1 rearrangements in non-small cell lung cancer: current insights and future directions. Cancer. (2025) 131:e35784. doi: 10.1002/cncr.35784. PMID: 40171848 PMC12551656

[18] Muminovic M Bato V Alvarez CA Raez LE . Importance of ROS1 gene fusions in non-small cell lung cancer. Cancer Drug Resist. (2023) 6:332–44. doi: 10.20517/cdr.2022.105. PMID: 37457125 PMC10344718

[19] Kim HR Lim SM Kim HJ Hwang SK Park JK Shin E . The frequency and impact of ROS1 rearrangement on clinical outcomes in never smokers with lung adenocarcinoma. Ann Oncol. (2013) 24:2364–70. doi: 10.1093/annonc/mdt220. PMID: 23788756

[20] Dyrbekk APH Grindstad T Skjefstad K Richardsen E Donnem T Kilvaer TK . Evaluation of ROS1 expression and rearrangements in a large cohort of early-stage lung cancer. Diagn Pathol. (2023) 18:70. doi: 10.21203/rs.3.rs-2021302/v1. PMID: 37237384 PMC10224579

[21] Boyle TA Masago K Ellison KE Yatabe Y Hirsch FR . ROS1 immunohistochemistry among major genotypes of non-small-cell lung cancer. Clin Lung Cancer. (2015) 16:106–11. doi: 10.1016/j.cllc.2014.10.003. PMID: 25467930 PMC4770803

[22] Chen W Jin D Shi Y Zhang Y Zhou Q Zhao J . The underlying mechanisms of lorlatinib penetration across the blood-brain barrier and the distribution characteristics of lorlatinib in the brain. Cancer Med. (2020) 9:4350–9. doi: 10.1002/cam4.3061. PMID: 32347012 PMC7300403

[23] Vallianatou T Strittmatter N Nilsson A Shariatgorji M Hamm G Pereira S . A mass spectrometry imaging approach for investigating how drug-drug interactions influence drug blood-brain barrier permeability. Neuroimage. (2018) 172:808–16. doi: 10.1016/j.neuroimage.2018.01.013. PMID: 29329980

[24] Akamine T Toyokawa G Tagawa T Seto T Takenoyama M Ichinose Y . Lorlatinib for the treatment of patients with non-small cell lung cancer. Drugs Today (Barc). (2019) 55:107–16. doi: 10.1358/dot.2019.55.2.2927983. PMID: 30816885

[25] Calles A Ponce S Navarro A Martinez-Marti A Felip E Nadal E . Efficacy and safety of lorlatinib in patients with ALK- and ROS1-rearranged metastatic non-small cell lung cancer treated within the compassionate use program in Spain. Cancer Treat Res Commun. (2025) 43:100905. doi: 10.1016/j.ctarc.2025.100905. PMID: 40154161

[26] Fabbri L Di Federico A Astolfi L Sperandi F Melotti B Ardizzoni A . From development to place in therapy of lorlatinib for the treatment of ALK and ROS1 rearranged non-small cell lung cancer (NSCLC). Diagnostics (Basel). (2023) 14:48. doi: 10.3390/diagnostics14010048. PMID: 38201357 PMC10804309

[27] Zhu VW Lin YT Kim DW Loong HH Nagasaka M Toi Y . An international real-world analysis of the efficacy and safety of lorlatinib through early or expanded access programs in patients with tyrosine kinase inhibitor-refractory ALK-positive or ROS1-positive NSCLC. J Thorac Oncol. (2020) 15:1484–96. doi: 10.1016/j.jtho.2020.04.019. PMID: 32360579

[28] Peled N Gillis R Kilickap S Froesch P Orlov S Filippi AR . GLASS: global lorlatinib for ALK(+) and ROS1(+) retrospective study: real world data of 123 NSCLC patients. Lung Cancer. (2020) 148:48–54. doi: 10.1016/j.lungcan.2020.07.022. PMID: 32799090

[29] Shaw AT Felip E Bauer TM Besse B Navarro A Postel-Vinay S . Lorlatinib in non-small-cell lung cancer with ALK or ROS1 rearrangement: an international, multicentre, open-label, single-arm first-in-man phase 1 trial. Lancet Oncol. (2017) 18:1590–9. doi: 10.1016/s1470-2045(17)30680-0. PMID: 29074098 PMC5777233

[30] Yang J Gong W . Lorlatinib for the treatment of anaplastic lymphoma kinase-positive non-small cell lung cancer. Expert Rev Clin Pharmacol. (2019) 12:173–8. doi: 10.1080/17512433.2019.1570846. PMID: 30657349

[31] Thummalapalli R Zhu VW White JR Negrao MV Reuben A Quek K . Lorlatinib tolerability and association with clinical outcomes in patients with advanced ALK- or ROS1-rearranged NSCLC: a brief report. JTO Clin Res Rep. (2023) 4:100546. doi: 10.1016/j.jtocrr.2023.100546. PMID: 37644967 PMC10460990

