# Supplementary material for: Case Report: Dynamic TKI combination strategies for EGFR-mutant NSCLC with acquired ROS1 fusion and brain metastases
Source: Front Oncol. 2026 Jun 4;16:1825642. doi: 10.3389/fonc.2026.1825642 (PMC13275228; doi:10.3389/fonc.2026.1825642)
Supplement: Supplementary file 1 [file Table1.docx]

# **CARE Checklist (2013) - EGFR-Mutant NSCLC with Acquired ROS1 Fusion Case Report**

****Article Title:**** Case Report: Dynamic TKI Combination Strategies for EGFR-Mutant NSCLC with Acquired ROS1 Fusion and Brain Metastases
****Authors:**** Hua Yang, Yong Da, Ge Shen, Qi-Yun Sun, Zhi-Yu Yao, Ya-Jing Zhou

| Item | Topic | No. | Checklist Item Description | Location in Manuscript |
| --- | --- | --- | --- | --- |
| ****1**** | ****Title**** | 1 | The diagnosis or intervention of primary focus followed by the words "case report" | ****Title line**** |
| ****2**** | ****Key Words**** | 2 | 2 to 5 key words that identify diagnoses or interventions in this case report, including "case report" | ****Keywords section**** |
| ****3**** | ****Abstract**** | 3a | Introduction: What is unique about this case and what does it add to the scientific literature? | ****Abstract sentences 1-2**** |
|  |  | 3b | Main symptoms and/or important clinical findings | ****Abstract sentence 3**** |
|  |  | 3c | The main diagnoses, therapeutic interventions, and outcomes | ****Abstract sentences 4-5**** |
|  |  | 3d | Conclusion—What is the main "take-away" lesson(s) from this case? | ****Abstract last sentence**** |
| ****4**** | ****Introduction**** | 4 | One or two paragraphs summarizing why this case is unique (may include references) | ****Introduction paragraph**** |
| ****5**** | ****Patient Information**** | 5a | De-identified patient specific information | ****Case Presentation - Diagnostic Assessment, sentence 1**** |
|  |  | 5b | Primary concerns and symptoms of the patient | ****Case Presentation - Diagnostic Assessment, sentences 1-2**** |
|  |  | 5c | Medical, family, and psycho-social history including relevant genetic information | ****Case Presentation - Diagnostic Assessment, sentence 1 (no smoking/family history)**** |
|  |  | 5d | Relevant past interventions with outcomes | ****Not applicable (initial diagnosis)**** |
| ****6**** | ****Clinical Findings**** | 6 | Describe significant physical examination (PE) and important clinical findings | ****Case Presentation - Diagnostic Assessment, detailed imaging findings**** |
| ****7**** | ****Timeline**** | 7 | Historical and current information from this episode of care organized as a timeline | ****Figure 2 (timeline portion) and Case Presentation treatment phase descriptions**** |
| ****8**** | ****Diagnostic Assessment**** | 8a | Diagnostic testing (such as PE, laboratory testing, imaging, surveys) | ****Case Presentation - Diagnostic Assessment, imaging and NGS/RT-PCR results at each stage**** |
|  |  | 8b | Diagnostic challenges (such as access to testing, financial, or cultural) | ****Not mentioned/Not applicable**** |
|  |  | 8c | Diagnosis (including other diagnoses considered) | ****Case Presentation - Diagnostic Assessment, clearly diagnosed as lung adenocarcinoma stage IVB**** |
|  |  | 8d | Prognosis (such as staging in oncology) where applicable | ****Case Presentation - Diagnostic Assessment, clearly stated as cT1c, cN3, cM1c (stage IVB)**** |
| ****9**** | ****Therapeutic Intervention**** | 9a | Types of therapeutic intervention (such as pharmacologic, surgical, preventive, self-care) | ****Case Presentation, detailed description of drug regimens and radiotherapy**** |
|  |  | 9b | Administration of therapeutic intervention (such as dosage, strength, duration) | ****Case Presentation, detailed drug dosages and radiotherapy parameters**** |
|  |  | 9c | Changes in therapeutic intervention (with rationale) | ****Case Presentation, each regimen change explained (progression/intolerance)**** |
| ****10**** | ****Follow-up and Outcomes**** | 10a | Clinician and patient-assessed outcomes (if available) | ****Case Presentation, imaging assessments; Patient Perspective section**** |
|  |  | 10b | Important follow-up diagnostic and other test results | ****Case Presentation, multiple NGS and pathology biopsy results**** |
|  |  | 10c | Intervention adherence and tolerability (How was this assessed?) | ****Case Presentation, detailed adverse reactions leading to dose reduction or discontinuation**** |
|  |  | 10d | Adverse and unanticipated events | ****Case Presentation, detailed adverse reactions for each drug**** |
| ****11**** | ****Discussion**** | 11a | A scientific discussion of the strengths AND limitations associated with this case report | ****Discussion, final paragraph (mentions limitations)**** |
|  |  | 11b | Discussion of the relevant medical literature with references | ****Discussion, in-depth discussion with literature citations throughout**** |
|  |  | 11c | The scientific rationale for any conclusions (including assessment of possible causes) | ****Discussion, discusses resistance mechanisms and possible drug interactions**** |
|  |  | 11d | The primary "take-away" lessons of this case report (without references) in a one paragraph conclusion | ****Discussion, final sentence**** |
| ****12**** | ****Patient Perspective**** | 12 | The patient should share their perspective in one to two paragraphs on the treatment(s) they received | ****Patient Perspective section**** |
| ****13**** | ****Informed Consent**** | 13 | Did the patient give informed consent? Please provide if requested | ****Informed Consent section**** |
